# *In vivo* Reprogramming of Cancer Metabolism by MYC

**DOI:** 10.3389/fcell.2017.00035

**Published:** 2017-04-11

**Authors:** Roman Camarda, Jeremy Williams, Andrei Goga

**Affiliations:** ^1^Department of Cell and Tissue Biology, University of California, San FranciscoSan Francisco, CA, USA; ^2^Biomedical Sciences Graduate Program, University of California, San FranciscoSan Francisco, CA, USA; ^3^Department of Medicine, University of California, San FranciscoSan Francisco, CA, USA; ^4^Helen Diller Family Comprehensive Cancer Center, University of California, San FranciscoSan Francisco, CA, USA

**Keywords:** MYC, metabolism, fatty acid oxidation, glutamine, glucose, transgenic cancer models, therapeutic irrigation, ras proteins

## Abstract

The past few decades have welcomed tremendous advancements toward understanding the functional significance of altered metabolism during tumorigenesis. However, many conclusions drawn from studies of cancer cells in a dish (i.e., *in vitro*) have been put into question as multiple lines of evidence have demonstrated that the metabolism of cells can differ significantly from that of primary tumors (*in vivo)*. This realization, along with the need to identify tissue-specific vulnerabilities of driver oncogenes, has led to an increased focus on oncogene-dependent metabolic programming *in vivo*. The oncogene c-MYC (MYC) is overexpressed in a wide variety of human cancers, and while its ability to alter cellular metabolism is well-established, translating the metabolic requirements, and vulnerabilities of MYC-driven cancers to the clinic has been hindered by disparate findings from *in vitro* and *in vivo* models. This review will provide an overview of the *in vivo* strategies, mechanisms, and conclusions generated thus far by studying MYC's regulation of metabolism in various cancer models.

## Introduction

Cancer is a disease of uncontrolled growth, and proliferating cells change their metabolic demands compared to quiescent cells (DeBerardinis et al., 2008; Vander Heiden et al., 2009). Tumor cells can outcompete normal cells, regardless of the proliferative capacity of the tissue of origin. Dysregulated metabolism is a hallmark of tumorigenesis (Hanahan and Weinberg, 2011), and such altered metabolism permits tumor cells to survive and proliferate despite adverse conditions.

Historical studies of altered metabolism in cancer pointed to increased glycolysis, and later glutaminolysis, as defining characteristic of tumor cells. Significant progress has been made studying glycolysis and glutaminolysis, and therapeutic targeting of these pathways is actively being pursued in the clinic (Altman et al., 2016; Hay, 2016). However, it has become increasingly apparent that while glycolysis and glutaminolysis certainly play major roles in some tumors (Hay, 2016; Altman et al., 2016), alternative sources of “fuel” can be just as, if not more, important (Cairns and Mak, 2016). Notably, targeting of alternative metabolic pathways, for example lipid biosynthesis, is currently in clinical trials against a variety of tumor types, and cannot be undervalued (Galluzzi et al., 2013).

**Figure 1:**
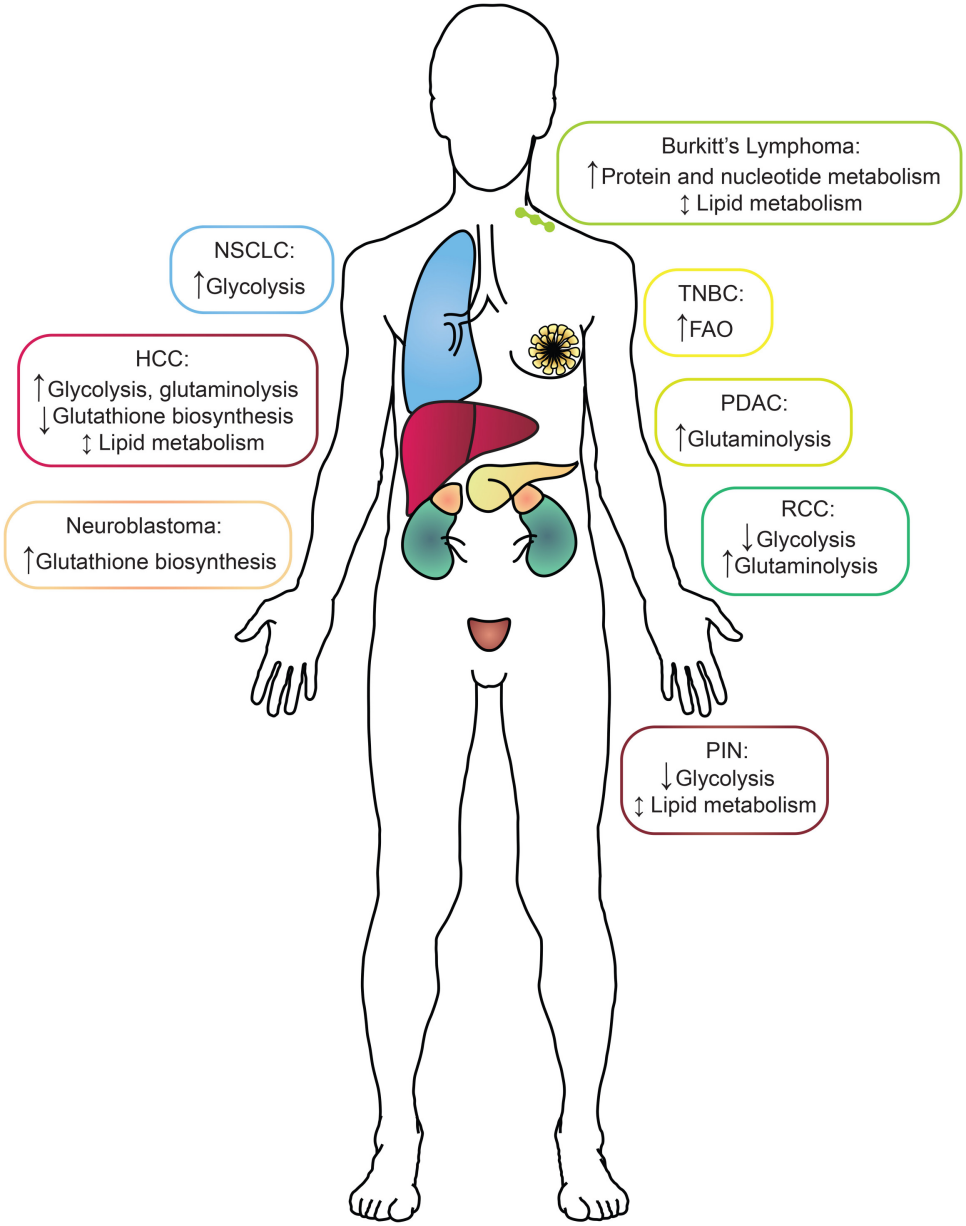
**A summary of the metabolic alterations found in each MYC-driven cancer type by tissue of origin**. Boxes surrounding each cancer indication are color-coded to match the tissue of origin. HCC, hepatocellular carcinoma; NSCLC, non-small-cell lung cancer; RCC, renal cell carcinoma; PDAC, pancreatic ductal adenocarcinoma; PIN, prostatic intraepithelial neoplasia; TNBC, triple-negative breast cancer.

A critical link between understanding cancer metabolism and targeting it therapeutically is identifying the upstream effectors that reshape tumor metabolism. The proto-oncogene MYC is a pleiotropic transcription factor and is one of the most commonly amplified or overexpressed genes in human cancers (Meyer and Penn, 2008). While MYC expression is dysregulated in a wide variety of cancers, it's oncogenic role has most thoroughly been studied *in vivo* in the context of transgenic models of aggressive breast, liver, lung, prostate, and kidney cancers, as well as neuroblastoma and lymphoma (see references below; Figure 1 and Table 1). For example, we and others have demonstrated that MYC expression is elevated in the estrogen, progesterone and human epidermal growth factor receptor-2 (HER2), receptor triple-negative subtype of breast cancer (TNBC; Horiuchi et al., 2012; Koboldt et al., 2012). Additionally, MYC translocation to the IgG locus plays a causal role in Burkitt's Lymphoma (Meyer and Penn, 2008; Eberlin et al., 2014). As a transcription factor, MYC's primary mode of transformation is through the pro-tumorigenic transcriptional dysregulation of a wide variety of processes including proliferation, cell size, apoptosis, and metabolism (Meyer and Penn, 2008). Regulation of MYC's transcriptional activity (Kress et al., 2015), and the role of MYC's transcriptional binding partners in the regulation of metabolism (Sloan and Ayer, 2010) have been studied and reviewed, and will not be discussed here. It is also important to note that given the broadly important role of MYC in cancer, a direct MYC inhibitor could be of great clinical utility. However, such an inhibitor has yet to be created, and the strategy of targeting MYC directly remains challenging (Lockwood et al., 2012; McKeown and Bradner, 2014). Thus, alternative strategies of targeting MYC-driven cancers via selective inhibition of cellular pathways, like metabolism, that may selectively kill MYC-overexpressing cells have attractive therapeutic potential. Indeed, the concept of specifically targeting metabolism to induce synthetic lethality in a MYC-dependent manner was pioneered by Shim et al. (1997), and expanded upon by many others (Yuneva et al., 2007; Dang, 2011).

**Table 1:** ***In vivo* transgenic models of MYC-driven cancer (excluding hydrodynamic models)**.

**Tissue/cancer specificity**	**Model**	**MYC-dependent metabolic pathways altered**
Liver—HCC	LAP-tTA/TRE-MYC	Glycolysis (Medina-Cleghorn and Nomura, 2014; Buescher et al., 2015), glutaminolysis (Buescher et al., 2015), glutathione biosynthesis (Altman et al., 2015), lipid metabolism (Bott et al., 2015)
Lung—NSCLC	SPC-rtTA/TRE-MYC	Glycolysis (Buescher et al., 2015)
Kidney—RCC	GGT-tTA/TRE-MYC	Glycolysis (Anderton et al., 2017), glutaminolysis (Anderton et al., 2017)
Pancreatic—PDAC	Pdx1-Cre/LSL-KRASG12D/R26-LSL-MYC	Glutaminolysis (Calvisi and Thorgeirsson, 2005)
Prostate—PIN	Pbsn-MYC	Glycolysis (Hu et al., 2011), lipid metabolism (Hu et al., 2011)
Neural crest—NB	TH-MYCN	Glutathione biosynthesis (Allen et al., 2011)
Lymphocytes—BL	Eμ-tTA/TRE-MYC	Lipid metabolism (Eberlin et al., 2014)
	Eμ-MYC/RPL24^+/−^	Protein metabolism (D'Cruz et al., 2001), nucleotide metabolism (Pfefferle et al., 2013)
Breast—TNBC	MMTV-rtTA/TRE-MYC	Fatty acid oxidation (Carter et al., 2016)

*A summary of the transgenic mouse models used thus far to study MYC-driven cancer metabolism in vivo. The tissue of origin, specific transgenes and primary altered metabolic pathway(s) studied in each model are noted. References for the models can be found in the main text. HCC, hepatocellular carcinoma; NSCLC, non-small-cell lung cancer; RCC, renal cell carcinoma; PDAC, pancreatic ductal adenocarcinoma; PIN, prostatic intraepithelial neoplasia; NB, neuroblastoma; BL, Burkitt's lymphoma; TNBC, triple-negative breast cancer*.

The ability of MYC to dynamically regulate cellular metabolism in cancer is well-established (Wahlström and Henriksson, 2015; Stine et al., 2015). However, it is important to note that many studies describing MYC's ability to reprogram tumor cell metabolism have been conducted *in vitro*, primarily using inducible/repressible transgenic and human cancer cell line models (Wahlström and Henriksson, 2015; Stine et al., 2015). While the importance and utility of *in vitro* cell culture models is undeniable, results from these models must be considered with caution when studying a process such as metabolism that is dependent on tumor cell environment (Mayers and Vander Heiden, 2015). Further, the dynamic nature of metabolic stressors and plasticity *in vivo* is difficult to model *in vitro*, particularly en masse. Primary tumors develop to form a complex tissue that is exposed to varying levels of oxygenation, and fluctuating concentrations of glucose, glutamine, amino acids and countless other metabolites that cannot be readily modeled in tissue culture (Mayers and Vander Heiden, 2015). Recent studies have also revealed an intimate connection between circadian rhythms and tissue-specific metabolism that has yet to be fully considered in the context of cancer metabolism (Abbondante et al., 2016). This last point is particularly prescient given the recent demonstration by Altman et al. that MYC itself can dysregulate circadian gene expression and metabolism (Altman et al., 2015), however, these findings have yet to be validated *in vivo*.

The disparate nature of *in vitro* and *in vivo* metabolism is exemplified by a recent study that took advantage of two transgenic mouse models of KRAS-driven non-small cell lung cancer (NSCLC; Davidson et al., 2016). Davidson et al. found that both models displayed increased utilization of glucose-derived carbon to fuel the tricarboxylic acid (TCA) cycle compared to normal lung *in vivo*, while neither tumor nor non-tumor utilized glutamine-derived carbon for the TCA cycle to a large extent. This is in stark contrast to a cell line derived from one of the transgenic models, which *in vitro* decreases its utilization of glucose for the TCA cycle and increases its utilization of glutamine to that end (Davidson et al., 2016). Thus, glutamine oxidation in this model system appears to be an artifact of the *in vitro* culture methods and is not observed *in vivo*. Such results thus raise doubt about the utility of targeting the glutamine pathway as a therapeutic target for primary KRAS-driven lung tumors.

Given the dynamic nature of MYC's function in diverse cellular contexts, and the potential for cell culture to confound our understanding of tumor metabolism, the goal of this review is to focus on the regulation of cancer metabolism by MYC *in vivo*. To clarify, our definition of *in vivo* refers to studies of metabolism with findings based on *de novo* MYC-driven tumorigenesis, usually in the form of transgenic mouse models. While we acknowledge that many findings from *in vitro* studies of MYC-driven cancer metabolism hold true *in vivo* (Wahlström and Henriksson, 2015; Stine et al., 2015), we will discuss here the various models used to study the regulation of cancer metabolism by MYC *in vivo* (summarized in Table 1), and provide broader context on some of the questions that remain to be answered.

## Use of transgenic mouse models and consideration of tissue-specific effects

The study of cancer metabolism *in vivo* is of course limited by the methods and unique challenges and considerations that the metabolism of complex tissues warrants (Mayers and Vander Heiden, 2015). One particularly important consideration is the difference between “snap-shot” strategies of studying metabolism vs. kinetic flux analyses, and how the use of chemically labeled metabolites factors into both approaches. The most common snap-shot method for studying metabolism is mass spectrometry-based metabolomics, which can be “targeted” for known metabolites or “untargeted” for unbiased detection of all metabolites present within a particular sample, and does not require any labeled metabolite (Medina-Cleghorn and Nomura, 2014). A second snap-shot strategy is ^13^C tracer analysis, in which a ^13^C-labeled metabolite is infused or fed to the subject, and mass spectrometry is used to identify downstream metabolite labeling patterns (Buescher et al., 2015). The use of ^13^C-labeled metabolites shifts from a snap-shot tracing study to a formal kinetic flux analysis when a much more complex series of considerations (metabolite uptake and secretion, as well as the kinetics of the biochemical reaction network to be probed) are taken into account (Buescher et al., 2015). A common approach to achieve flux analysis is with constant infusion of an isotopically labeled tracer, ^13^C-glucose for example, that will achieve isotopic steady state as ^13^C enrichment becomes stable over time (Buescher et al., 2015). Understanding the differences between these methods, and the conclusions that can be drawn from them, is vital. In particular, snap-shot metabolomics is often used to prematurely draw conclusions about the activity of a metabolic pathway, when the elevation or decrease of a particular metabolite does not necessarily reflect activation or inhibition of an entire pathway (Medina-Cleghorn and Nomura, 2014; Buescher et al., 2015). Moreover, interpretation and validation of metabolic data is critical, as for example, accumulation of a particular metabolite could have multiple potential interpretations (i.e., increased activity of an upstream anabolic pathway or decreased activity of a downstream catabolic pathway). An important caveat to the study of *in vivo* metabolism is that tumor tissue is often analyzed at a bulk level, and as the work of Aran et al. and many others has demonstrated, the composition of solid tumors includes a number of different cell types (Aran et al., 2015) whose metabolism is rarely accounted for in such bulk analyses.

With a cadre of strategies in hand, the study of cancer metabolism *in vivo* then becomes a function of the models or the clinical samples available for analysis. In this section, we will address some of the most thoroughly used models to study the metabolism of MYC-driven cancer (Table 1). The overall message is that while MYC-driven metabolism during tumorigenesis is quite tissue-specific, some shared pathways also emerge (Figure 1).

## MYC dysregulates glucose and glutamine metabolism

In hepatocellular carcinoma (HCC), MYC is found to be frequently amplified and/or overexpressed, and is associated with poorly differentiated tumors and poor prognosis (Shachaf et al., 2004; Calvisi and Thorgeirsson, 2005; Kaposi-Novak et al., 2009; Lim et al., 2014; Anderton et al., 2017). In addition, MYC expression is commonly found to be upregulated in hepatoblastoma (HB), a liver tumor type that predominates in pediatric patients (Wang et al., 2016).

To study MYC-dependent metabolism in HCC, we and others have utilized the MYC-driven LAP-tTA/TRE-MYC (LT2-MYC) transgenic mouse model of liver cancer initially developed in the lab of J.M. Bishop, which allows for MYC overexpression specifically in hepatocytes in the absence of doxycycline (Shachaf et al., 2004). Importantly, mRNA expression analysis reveals that LT2-MYC tumors effectively model poorly differentiated, aggressive liver cancer (Lim et al., 2014). Using this model, we probed for changes in glycolytic metabolism using hyperpolarized ^13^C-pyruvate magnetic resonance spectroscopic imaging (MRSI) during *de novo* tumorigenesis and inducible tumor regression. More specifically, hyperpolarized ^13^C-pyruvate MRSI allows for *in vivo* flux analysis of pyruvate to lactate and/or alanine conversion. With this modality, we found that MYC induction led to increased pyruvate to alanine conversion in the liver that preceded overt tumor formation, while full-blown tumors displayed increased pyruvate to lactate conversion. Both of these phenotypes were reversed during tumor regression. Mechanistically, mRNA expression analysis revealed coordinate changes in the levels of TCA cycle and glycolytic enzymes that supported the observed metabolic changes. In particular, there was a specific elevation of glutamate pyruvate transaminase 1, which converts pyruvate to alanine, in pre-tumorigenic liver, while lactate dehydrogenase A (LDHA), which converts pyruvate to lactate, was specifically upregulated in tumors (Hu et al., 2011). Studies such as this indicate that imaging of downstream glycolysis pathway events can identify the earliest stages of tumor formation and regression and that these metabolic changes are indeed MYC dependent.

The notion that MYC drives increased glycolysis in liver cancer was further bolstered by a parallel study of MYC-driven metabolism using the same LT2-MYC model. Yuneva et al. utilized a combination of steady-state profiling techniques including nuclear magnetic resonance spectroscopy with or without ^13^C-glucose and ^13^C-glutamine labeling, as well as ^18^F-fluorodeoxyglucose positron emission tomography and mass spectrometry (Yuneva et al., 2012). The authors found that MYC-driven liver tumors displayed increased glucose uptake and catabolism to lactate and TCA cycle intermediates, as well as increased glutamine catabolism to support the TCA cycle. These findings were supported by increased expression of LDHA, hexokinase 2 (Hk2), and glutaminase 1 (Gls1), and decreased expression of glutamine synthetase (Glul). The importance of glutamine catabolism in MYC-driven HCC was further demonstrated by Xiang et al. who demonstrated that genetic ablation of one copy of Gls1 or treatment with two different inhibitors of Gls1 could significantly prolong survival in this same model (Xiang et al., 2015). This was in direct contrast to glucose and glutamine utilization in transgenic MYC-driven lung tumors (Wang et al., 2001; Allen et al., 2011). Unlike MYC-driven liver tumors, MYC-driven lung tumors displayed elevated lactate and glutamine levels, which was suggestive of increased glucose catabolism, but not glutamine catabolism. MYC-driven lung tumors displayed increased LDHA, Hk2, Gls1 as well as Glul. Likewise, a similar model of transgenic MYC-driven lung cancer displayed increased LDHA and Hk2, as well as enzymes from several other metabolic pathways, at the mRNA level (Ciribilli et al., 2016).

Although MYC pathway activation is elevated in the majority of renal cell carcinoma (RCC) cases, a formal study of MYC's role in the pathogenesis and the metabolism of RCC had been lacking. To study MYC in RCC, Shroff et al. created an inducible transgenic model of renal cell carcinoma (GGT-tTA/TRE-MYC) in which MYC is specifically overexpressed in the kidney in the absence of doxycycline (Shroff et al., 2015). Using desorption electrospray ionization mass spectrometry imaging (DESI-MSI), the authors studied the metabolic profiles of non-tumor kidney, MYC-driven kidney tumors at 2 and 4 weeks post-MYC induction, and regressed tumors after 4 weeks of switching MYC off. The authors noted multiple metabolic changes, including in the relative abundance of a variety of long-chain fatty acids in tumors compared to non-tumor kidneys and regressed tumors. Schroff and colleagues focused on glutamine metabolism after mRNA expression analysis revealed a downregulation in many glycolytic genes, but an upregulation in genes associated with glutaminolysis. The authors confirmed that glutamate and TCA cycle intermediates were elevated in tumors using DESI-MSI, and found that their transgenic tumors, as well as MYC^high^ human RCC, stained positively for Gls1 and Gls2, suggesting an elevation in glutaminolysis. Decreased staining of the transgenic tumors for Hk1 and LDHA further supported diminished glycolysis. Finally, the authors found that pharmacological inhibition of Gls1 with bis-2-(5-phenylacetamido-1,2,4-thiadiazol-2-yl) ethyl sulfide (BPTES) abrogated the growth of MYC-driven kidney tumors (Shroff et al., 2015), implicating glutamine utilization as critical for MYC-driven RCCs, similar to what was found in MYC-driven HCC (Xiang et al., 2015).

Additional evidence that MYC dysregulates glutamine metabolism was provided by a recent study that found elevation of the glutamine synthetase (Glul) enzyme and glutamine abundance in a transgenic mouse model of dual MYC and KRAS-driven pancreatic cancer, compared to tumors driven by KRAS alone (Bott et al., 2015). These studies suggested Glul is induced by MYC. Further support that MYC dysregulates glucose metabolism was provided when mass spectrometry-based metabolomic analysis was used to compare the metabolic profiles of established transgenic mouse models of MYC- or AKT-driven prostate cancer (Ellwood-Yen et al., 2003; Majumder et al., 2003), as well as human prostate cancer samples that had been profiled for activated phospho-AKT and MYC levels. The authors found coordinately decreases in glucose-related metabolites and downregulation of HK2 and the glucose transporter GLUT-1 in mouse and human prostate tumors that were MYC^high^, compared to control tissue and AKT^high^ tumors. In addition, the authors found specific dysregulation of several long-chain fatty acids in MYC^high^ tumors, but the functional significance of these changes was not addressed (Priolo et al., 2014).

In summary, the ability of MYC to alter glucose and glutamine metabolism in cancer is clear. However, the studies of MYC-driven liver, lung, kidney, pancreatic, and prostate cancers studied above highlight the fact that MYC can up- or down-regulate either or both of these pathways depending on tissue context. Furthermore, Shroff et al. were the only group to formally demonstrate that dysregulation of one of these pathways leads to a reliance upon it that may have therapeutic potential (Shroff et al., 2015). Further, studies in the remaining cancer types discussed above will be necessary to determine if targeting glucose or glutamine metabolism will have therapeutic utility.

## MYC regulates downstream glutamine utilization

**Figure 2:**
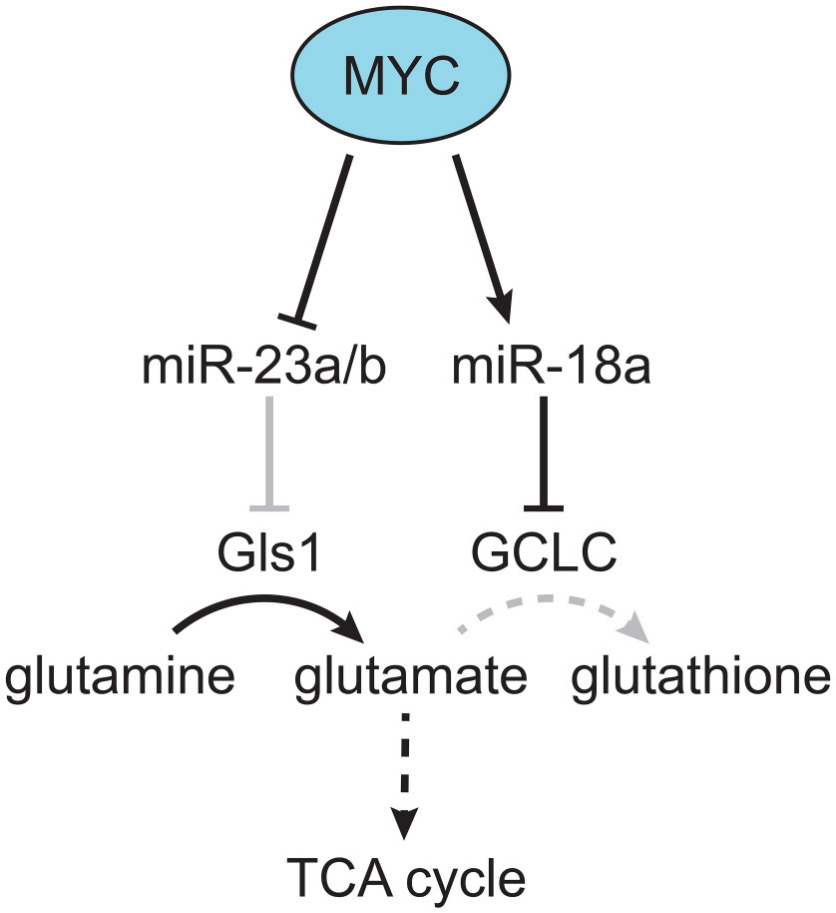
**MYC-dependent miRNA regulation of glutamine metabolism**. MYC was found to downregulate miR-23a/b, which targets Gls, resulting in increased production of glutamate from glutamine (Gao et al., 2009). In addition, MYC was found to upregulate miR-18a, which targets GCLC, resulting in decreased production of glutathione from glutamate, and increased flow of glutamine-derived carbon into the TCA cycle (Anderton et al., 2017). Gray lines indicate a decreased effect, and dotted lines indicate a multi-step metabolic pathway.

Although the LT2-MYC model had multiple changes in glucose and glutamine metabolism (Hu et al., 2011; Yuneva et al., 2012), other metabolic pathways had not been fully explored. Using the conditional MYC-driven liver cancer model we conducted global mRNA expression and mass spectrometry-based metabolomic analyses on LT2-MYC tumors vs. control uninduced transgenic livers (Anderton et al., 2017). Using an integrated bioinformatics approach, we probed for metabolic pathways coordinately dysregulated in both transcript and metabolite levels. Of the six pathways identified: glutathione metabolism; glycine, serine, and threonine metabolism; aminoacyl-tRNA biosynthesis; cysteine and methionine metabolism; ABC transporters; and mineral absorption, we focused on glutathione metabolism (Anderton et al., 2017). We found a marked decrease in the reduced and oxidized form of glutathione, as well as the enzyme responsible for *de novo* glutathione biosynthesis, glutamate-cysteine ligase, catalytic subunit (GCLC). Because glutathione is synthesized downstream of glutamine conversion to glutamate, we performed mass spectrometry-based tracing analysis with ^13^C-glutamine in a somatic transgenic model of MYC-driven liver cancer (Tward et al., 2005). We found that glutamine-derived carbons preferentially fueled the TCA cycle vs. glutathione production in MYC-driven liver tumors compared to control liver tissue. Mechanistically, we found that GCLC expression was downregulated by miR-18a in a MYC-dependent manner. Treatment of LT2-MYC tumor-bearing mice with a locked-nucleic acid antagonist of miR-18a significantly rescued GCLC expression and glutathione levels *in vivo*. In addition, miR-18a was significantly elevated in human HCC compared to non-tumor liver, was negatively correlated with GCLC expression in human HCC, and was positively correlated with alpha-fetoprotein (AFP) expression, which is associated with aggressive liver cancer. Finally, we found that LT2-MYC tumors displayed increased sensitivity to an oxidative stress inducer, diquat, compared to non-tumor liver. In particular, diquat-treated tumors displayed a specific and significant increase in cell loss, TUNEL staining as a marker of apoptosis, and decreased MYC expression (Anderton et al., 2017). Notably, it had been previously demonstrated *in vitro* that MYC-dependent suppression of miR-23a/b results in increased Gls1 and glutaminolysis activity (Gao et al., 2009; Kota et al., 2009). Thus, MYC can alter the expression of specific miRNAs (i.e., miR18a and miR23a) which in turn regulate glutamine metabolism. MYC-dependent regulation of miRNAs may be a common mechanism through which MYC reprograms tumor metabolism (Figure 2) and deserves broader consideration beyond HCC.

In neuroblastoma, the MYC-related transcription factor MYCN is found to be amplified in ~20% of neuroblastomas, and its amplification is associated with poor prognosis (Carter et al., 2016). To study the role of MYCN in neuroblastoma metabolism, Carter et al. utilized the TH-MYCN transgenic model of MYCN-driven neuroblastoma in which MYCN is overexpressed in cells of the neural crest (Weiss et al., 1997). Using mass spectrometry-based metabolomics, the authors performed global metabolic profiling of MYCN-driven neuroblastoma at multiple time-points representing hyperplastic ganglia, early tumors, and advanced tumors. Grouping the metabolomic data into pathway analysis, it was found that glutathione metabolism was the most significantly dysregulated pathway, with all metabolites associated with glutathione biosynthesis elevated in MYCN-driven tumors compared to control ganglia. Interestingly, the majority of enzymes associated with glutathione biosynthesis, including GCLC, were found to be downregulated at the mRNA level. Therefore, the authors speculated that increased protein biosynthesis, which was evidenced by a significant increase in the expression of ribosome biogenesis genes, was responsible for the observed increase in glutathione, although this contention was not formally tested. Regardless, the increase in glutathione led the authors to hypothesize that MYCN-driven neuroblastoma could have an increased dependence upon glutathione metabolism. Indeed, the authors found that BSO, an inhibitor of GCLC, could reduce sympathetic ganglia hyperplasia and delay tumor onset when given prophylactically. In addition, GCLC inhibitors did not have an effect on the growth of established tumors when given alone, but did have a significant benefit when given with the clinically relevant chemotherapeutic agent vincristine, compared to BSO alone or vincristine alone (Carter et al., 2016). Thus, in both MYC driven liver and neuroblastoma models GCLC expression is suppressed, though the effects on glutathione production appear to be contextually dependent. We postulate that in the setting of low GCLC expression, and consequently low GSH production that some MYC-driven tumors, such as liver cancers, may be especially sensitive to exogenous oxidative stress (Anderton et al., 2017).

Terunuma et al. conducted mass spectrometry-based metabolomics on primary breast cancer samples and adjacent non-tumor tissue (Terunuma et al., 2014). The authors found a number of differences in metabolite abundance between tumor and non-tumor samples, and probed further into the differences between ER-positive and ER-negative tumors as well as tumors from individuals with African ancestry vs. European ancestry. The authors chose to focus on 2-hydroxyglutarate (2-HG), a known “oncometabolite,” which was found to be preferentially elevated in ER-negative tumors. Interestingly, 2-HG accumulation normally occurs in the context of isocitrate dehydrogenase (IDH) 1 or 2 mutation, but the authors did not find evidence of IDH mutation in breast cancer. It was recently demonstrated that 2-HG can be produced via LDHA in the context of hypoxia (Intlekofer et al., 2015; Oldham et al., 2015), but Terunuma et al. did not address whether hyoxia could explain 2-HG production in the breast tumors analyzed. However, they did find a strong correlation between 2-HG levels, MYC pathway activity, glutaminolysis-associated metabolites, and Gls1 expression. Further, the authors provided *in vitro* evidence that 2-HG production occurs during glutamine catabolism, and that MYC is both necessary and sufficient for elevated 2-HG levels (Terunuma et al., 2014) in breast cell lines. These data suggest that MYC, albeit via a yet unclear mechanism, is able to promote glutamine utilization for 2-HG production in cancer. It is of course tempting to speculate that MYC-dependent regulation of LDHA, as discussed above, may contribute to the 2-HG production observed, but this remains to be determined.

In summary, MYC's regulation of glutamine metabolism is extensive. In the case of glutathione, relative decreases (Anderton et al., 2017) and increases (Carter et al., 2016) were observed depending on the cancer type. With a decrease, tumors were found to be sensitive to an inducer of oxidative stress (Anderton et al., 2017), while an increase led the tumors to be sensitive to GCLC inhibition during the early phase of tumor formation (Carter et al., 2016). Interestingly, in neuroblastoma the elevation of glutathione occurred despite a downregulation in GCLC mRNA levels. It would be interesting to determine if the decrease in GCLC observed in neuroblastoma is miR-18a-dependent. An alternative downstream use of glutamine to generate 2-HG has also been postulated in primary breast cancers. It remains unclear how MYC activity could induce 2-HG production, thus the therapeutic utility and potential to target this pathway have not been explored (Terunuma et al., 2014).

## MYC dysregulates lipid metabolism

The role of MYC in HB metabolism has not been studied as extensively as HCC (Cairo et al., 2008), but it is worth noting that a recent study performed global mRNA expression analysis in a somatic transgenic model of β-catenin/YAP-driven HB performed in mice with either MYC-wildtype (WT) or MYC-knockout (KO) hepatocytes. The authors found that MYC promoted tumor progression, but not initiation, and were able to identify several metabolic pathways with differential enzyme expression and pathway activity in MYC-WT vs. MYC-KO tumors (Wang et al., 2016). For example, MYC-KO tumors displayed reduced expression of the fatty acid transporter CD36, with a concomitant decrease in lipid droplet levels and fatty acid oxidation (FAO; Wang et al., 2016). Given these results in HB, it would be interesting to determine if MYC also dysregulated lipid metabolism in HCC. To that end, Perry et al. utilized DESI-MSI to not only detect differential abundance of lipid species in non-tumor liver, early LT2-MYC tumors, late tumors, and regressed tumors, but also generate a spatial localization of the detected lipids with ~200 μm resolution (Perry et al., 2013). The authors found that a number of lipid species displayed differential abundance in tumor vs. non-tumor tissue, but did not pursue the functional significance of these changes.

The work of Perry et al. in MYC-driven liver cancer later led the same lab to use DESI-MSI to study MYC-driven lymphoma. MYC is known to be broadly dysregulated in aggressive lymphomas, and in Burkitt's lymphoma the MYC gene is translocated next to the immunoglobulin heavy chain enhancer in virtually all cases (Meyer and Penn, 2008; Eberlin et al., 2014). To study MYC-driven lymphoma Eberlin et al. utilized the conditional Eμ-tTA/TRE-MYC transgenic mouse model in which MYC is specifically expressed in lymphocytes only in the absence of doxycycline (Felsher and Bishop, 1999). The authors reported a number of lipids that displayed differential abundance in MYC-driven lymphoma compared to control non-tumor thymus. In addition, the authors performed DESI-MSI on 15 human lymphoma samples, including five cases of Burkitt's lymphoma, that were profiled for MYC expression such that they were classified as MYC^high^ or MYC^low^. Interestingly, there were many similarities between the lipid profiles of the mouse MYC-driven lymphomas and the human MYC^high^ lymphomas, and both were distinct from the human MYC^low^ lymphomas (Eberlin et al., 2014). In addition, some of the most differentially increased lipids in MYC-driven lymphomas were multiple cardiolipin species, which are known to play critical roles in mitochondrial membrane integrity. Thus, although Eberlin et al. did not pursue the functional significance of dysregulated lipid metabolism, these changes could support alternative aspects of MYC-driven metabolism in lymphoma. Additionally, it is interesting that Eberlin *et al* acknowledge in their discussion a potential relationship between altered lipid abundance and FAO, and a separate study indeed found that inhibition of FAO was able to significantly delay tumorigenesis in a constitutive model of transgenic MYC-driven lymphoma (Eμ-MYC; Harris et al., 1988; Pacilli et al., 2013).

As mentioned above, we and others have demonstrated that MYC expression is disproportionately elevated in TNBC compared to receptor-positive (RP) tumors (Horiuchi et al., 2012; Koboldt et al., 2012). Thus, we were particularly interested in the use of the MYC-driven MMTV-rtTA/TRE-MYC (MTB-TOM) transgenic mouse model of breast cancer, in which MYC is overexpressed specifically in mammary epithelial cells in a doxycycline-inducible manner (D'Cruz et al., 2001). It is important to note that while MYC is certainly overexpressed in this model, which mimics the clinically observed increase in MYC expression in TNBC, it was also confirmed by unbiased clustering of mRNA expression analysis that the MTB-TOM model does resemble the Basal/TN subtype of breast cancer (Pfefferle et al., 2013). Using this model, we performed steady-state metabolomics and ^13^C-tracing analysis and found that FAO was dysregulated. We then used a ^14^C-oleic acid oxidation assay to confirm that FAO was elevated specifically in MYC-overexpressing TNBC. Given the elevation in FAO, a pathway known to fuel the TCA cycle and ATP production, we hypothesized this pathway could be required to fuel bioenergetic metabolism in MYC-overexpressing TNBC, and could have therapeutic potential. To address this hypothesis in a more clinically relevant model, we utilized a recently described panel of breast cancer patient-derived xenografts (PDX; DeRose et al., 2011). Using a specific inhibitor of the FAO pathway, etomoxir, we found that inhibition of FAO decreased bioenergetic metabolism and inhibited tumorigenesis in a MYC^high^ TN PDX, but did not inhibit tumorigenesis in a MYC^low^ TN PDX model (Camarda et al., 2016). Notably, a separate study found elevated FAO in TNBC, and described an additional downstream role for FAO in promoting autophosphorylation and activation of the oncogenic Src kinase (Park et al., 2016). It remains to be seen whether or not there is a functional interaction between MYC and Src in TNBC, and whether Src could be a mechanism of FAO upregulation in MYC-driven TNBC, or vice versa. In addition, as mentioned above, Terunuma et al. found elevation of acyl-carnitines, the bottleneck intermediate of FAO, in ER-negative human tumors compared to ER-positive or non-tumor tissue (Terunuma et al., 2014), supporting our findings of dysregulated FAO in TNBC (Camarda et al., 2016).

Although several studies have now indicated that MYC is capable of dysregulating lipid metabolism, and in particular FAO, no study has yet to validate a downstream mechanism by which MYC activation dysregulates lipid metabolism and/or FAO *in vivo*. It is worth noting that several potential mechanisms have been described *in vitro*, including MYC-dependent induction of mitochondrial biogenesis (Li et al., 2005), which has been functionally linked to FAO in the context of MYC inhibition (Zirath et al., 2013). In addition, there are several other hypotheses supported by the literature that are worth noting. First, we found a marked downregulation in acetyl-CoA carboxylase 2 (ACC2) protein expression in MYC^high^, but not MYC^low^ PDXs, and it has been demonstrated that downregulation of ACC2 in transgenic mice is sufficient to upregulate FAO *in vivo* (Abu-Elheiga et al., 2001). Second, fatty acid binding proteins (FABPs) are known to play a supporting role in fatty acid oxidation as they are responsible for trafficking fatty acids throughout the cell (Nieman et al., 2011). In ovarian cancer that metastasizes to the omentum it was demonstrated that FABP4 is upregulated in tumor cells and expressed in omental adipocytes, and is necessary in both cell types to support metastatic tumorigenesis (Nieman et al., 2011). Furthermore, FABP5 has been found to be upregulated in TNBC, and is associated with poor prognosis and recurrence-free survival in TNBC (Liu et al., 2011). Thus, we postulate that MYC reprograms lipid metabolism in TNBCs via coordinated suppression of fatty acid synthesis and upregulation of oxidation to support tumor metabolic demands.

Finally, we and others recently described the necessity for PIM kinase activity in MYC-overexpressing TNBC (Brasó-Maristany et al., 2016; Horiuchi et al., 2016). PIM expression can promote PGC1α expression, a master regulator of FAO (Beharry et al., 2011). In addition, a recent study suggests that there may be functional redundancy between PIM and PI3K in breast cancer, and because PI3K is a known regulator of glycolysis, PIM may then play a role in regulation of glycolysis in MYC-overexpressing TNBC (Hu et al., 2016; Le et al., 2016). Further studies are necessary to determine which, if any, of these potential mechanisms are indeed at play in the regulation of FAO in MYC-overexpressing TNBC.

In summary, MYC is capable of dysregulating lipid metabolism in multiple cancer types, but a mechanism has yet to described. Given that our work found that inhibition of FAO is a therapeutic strategy against MYC-overexpressing TNBC (Camarda et al., 2016), and a separate study found similar results in a model of MYC-driven lymphoma (Pacilli et al., 2013), it will be interesting to determine if this strategy could be expanded to MYC-driven HB and/or HCC.

## Studies of protein and nucleotide metabolism in MYC-driven lymphoma

In addition to studies of lipid metabolism, the Eμ-MYC model has also been used for studies of protein and nucleotide metabolism. Eμ-MYC lymphomas display elevated protein translation, a common of feature of many cancer types (Barna et al., 2008). Barna et al. created a bi-allelic model in which haploinsufficiency of the ribosomal protein RPL24 results in reduced protein translation back to non-tumor levels. When this model was bred to the Eμ-MYC model it resulted in decreased tumorigenesis (Barna et al., 2008). With this model, the same lab recently utilized NMR-based metabolomic analysis to profile changes in a number of metabolic pathways in non-tumor lymphocytes, pre-tumor MYC-driven lymphocytes, lymphocytes with reduced translation, MYC-driven lymphocytes with normalized translation, and tumorigenic MYC-driven lymphocytes. Cunningham et al. found that the most notable translation-dependent difference detected was a reduction in nucleotide-related metabolites, specifically inosine monophosphate and adenosine mono-, di-, and triphosphate. The authors then demonstrated that a single enzyme, phosphoribosyl-pyrophosphate synthetase 2 (PRPS2), is responsible for increased nucleotide metabolism in MYC-driven lymphoma via a cis-regulatory element in its 5′ UTR that is activated by translation initiation factor eIF4E, which is itself hyperactivated in tumors. Additionally, MYC-driven lymphomagenesis is at least in part dependent upon PRPS2 as Eμ-MYC crossed with PRPS2-null mice have a significant delay in tumor initiation as well as a significant increase in survival (Cunningham et al., 2014). Interestingly, elevated protein synthesis in this model has also been linked to increased activation of the unfolded protein response, which ultimately promotes tumor cell survival via autophagy (Hart et al., 2012). Thus, a combined increase in translation and autophagy may contribute to MYC-driven metabolic adaptation in lymphomas.

## Regulation of MYC by metabolism

While MYC reprograms metabolism, there is also mounting evidence of metabolic regulation of MYC in cancer and tissue homeostasis. One notable example is the regulation of MYC protein levels by HMG-CoA reductase, which has been demonstrated in the Eμ-tTA/TRE-MYC model of lymphoma, as well as the LT2-MYC model of liver cancer (Shachaf et al., 2007; Cao et al., 2011). Mechanistically, HMG-CoA reductase inhibition via atorvastatin reduced RAS and ERK1/2 signaling in lymphoma, resulting in decreased ERK-dependent MYC phosphorylation, and reduced MYC levels (Shachaf et al., 2007). In liver cancer, however, atorvastatin was found to decrease MYC phosphorylation and protein levels downstream of Rac GTPase activity (Cao et al., 2011). The broader implication of this finding is that a HMG-CoA reductase inhibitor such as atorvastatin deserves further consideration in MYC-overexpressing tumor types, and indeed atorvastatin did have anti-tumorigenic activity in the aforementioned models of MYC-driven liver cancer and lymphoma (Shachaf et al., 2007; Cao et al., 2011). A second example is the regulation of MYC protein levels by the enzyme O-linked N-acetylglucosamine transferase (OGT), which catalyzes post-translational O-GlcNAcylation of proteins. This phenomenon was demonstrated in a transgenic mouse model of liver cancer with elevated OGT activity (Burén et al., 2016). Interestingly, it has been previously demonstrated that MYC can be glycosylated on threonine 58, a key regulatory residue that is also phosphorylated, but the functional significance of this modification remains to be elucidated (Chou et al., 1995).

## Studying MYC and metabolism in human patients

While transgenic and PDX mouse models are invaluable in studying the role of MYC in cancer metabolism, the ultimate goal of these studies is to translate the findings from mouse models to the clinic. The study of cancer metabolism in the clinic has actually been a common practice for more than two decades via the use of the glucose analog ^18^F-fluorodeoxyglucose (FDG; Fletcher et al., 2008). Specifically, intravenous injection of ^18^F-FDG coupled with positron emission tomography (PET) allows for the imaging of a variety of tumor types, which preferentially take up glucose to a higher degree than most non-tumor tissues (Fletcher et al., 2008). Upregulation of hexokinase, which is very likely a MYC transcriptional target in at least some tumor tissues given it's strong MYC-dependent regulation as discussed above and elsewhere (Kim et al., 2007), results in phosphorylation and trapping of the FDG probe in cancers (Fletcher et al., 2008). Although ^18^F-FDG-PET imaging has generally been used to detect tumors, recent advancements in our understanding of the biology of tumorigenesis have led to much more specific uses for ^18^F-FDG-PET. For example, Palaskas et al. reasoned that a correlation between the expression of some mRNAs and ^18^F-FDG uptake may allow ^18^F-FDG-PET to identify the driver oncogene(s) or oncogenic pathway(s) active in a patient's tumor. The authors integrated mRNA expression analysis and ^18^F-FDG uptake from a panel of cancer cell lines and 18 patients with breast cancer. Gene set enrichment analysis revealed a number of upregulated molecular pathways in the cell lines and patients with higher ^18^F-FDG uptake including, not surprisingly, glycolysis. The authors then probed further for associations between the ^18^F-FDG signature and breast cancer subtypes and molecular drivers, and found that the ^18^F-FDG signature correlated best with the TN/basal subtype and MYC-dependent transcriptional activity. In addition, the authors retrospectively stained biopsies from the 18 breast cancer patients, and found a significant increase in MYC protein staining of the tumors with high ^18^F-FDG uptake (Palaskas et al., 2011). To our knowledge, further studies correlating MYC expression with ^18^F-FDG uptake in human tumors have not been conducted, but should be of further consideration.

Although glucose uptake measurement via ^18^F-FDG-PET is an invaluable clinical tool, some tumors are inherently ^18^F-FDG-negative (Fletcher et al., 2008). Likewise, some non-tumor tissues demonstrate high glucose ultilization (i.e., brain and liver), making discernment of tumors via ^18^F-FDG-PET challenging. Thus, alternative metabolites with high avidity for certain tumor types are needed. To that end, preclinical studies have been performed in the MTB-TOM MYC-driven breast cancer model with ^18^F-(2S,4R)4-fluoroglutamine (Lieberman et al., 2011), which could be useful in a number of MYC-driven tumors that upregulate glutaminolysis as discussed above. In addition, acetate was recently described by two studies as a critical carbon fuel for a variety of primary tumors and tumors that have metastasized to the brain (Comerford et al., 2014; Mashimo et al., 2014). The critical acetate oxidation enzyme in cancer appears to be the acetyl-CoA synthetase enzyme ACSS2, which was found to be essential for tumorigenesis in a MYC-driven model of liver cancer, and increased expression of ACSS2 was associated with poor prognosis in TNBC (Comerford et al., 2014). Notably, this study that focused on both primary brain tumors and tumors that metastasized to the brain. Four patients were infused with [1,2-^13^C]acetate during surgical resection of their tumors. Post-operative NMR revealed *de novo* oxidation of acetate to fuel the TCA cycle (Mashimo et al., 2014). Thus, acetate deserves broader consideration as a bioenergetics substrate in MYC-overexpressing tumors, both in terms of therapeutic targeting and for imaging purposes. Finally, hyperpolarized 1-^13^C-pyruvate MRSI has been used pre-clinically (Hu et al., 2011), but has also been adopted for imaging of patient tumors as part of a first-in-man clinical trial (Nelson et al., 2013). Indeed, there is clear interest and opportunity for this modality to enter the clinic, especially with expanded probes beyond 1-^13^C-pyruvate, which so far has been the most well-studied (Kurhanewicz et al., 2011).

Finally, it is worth taking note of several studies that focused almost entirely on the analysis of clinical samples. Importantly, these studies did not make a functional connection between the metabolic phenotypes observed and MYC activity, even though MYC has established functional roles in the cancer types studied. For those interested, we refer to metabolic profiling performed on primary tumors and serum samples from patients with HCC (Huang et al., 2013; Liu et al., 2014), as well as breast cancer (Cao et al., 2014; Kanaan et al., 2014; Cui et al., 2016). In addition, integrated metabolomic and proteomic analysis has been performed on primary RCC tumors (Wettersten et al., 2015).

## Broader implications

In this review, we have focused on the role of MYC in regulating cancer metabolism *in vivo*. The majority of studies to date have used transgenic mouse models or primary tumors. Thus, there remains a tremendous amount of work to be done looking outside the confines of the primary tumor to the role of MYC in metastatic tumors, as well as cells within the microenvironment and non-adjacent normal tissue, both of which will ultimately have tremendous influence on which therapeutic strategies can be translated to the clinic. With respect to metastasis, we recently performed single-cell mRNA expression analysis on *de novo* low- and high-burden metastases from orthotopic TNBC PDXs and found that MYC expression was significantly elevated in high-burden metastases (Lawson et al., 2015). Given that cancer cell metabolism has been shown to change in metastasis initiating cells (Pascual et al., 2016), when the metastatic cells are in circulation (LeBleu et al., 2014), and depending on which organ the metastatic tumor colonizes (Dupuy et al., 2015), further studies will need to determine whether the reliance on FAO, glucose, glutamine or other metabolites present in primary MYC-overexpressing TNBC is maintained in high-burden metastases.

Another aspect of the microenvironment that deserves significant consideration is the immune cell component. A recent study demonstrated that tumors with elevated glucose consumption effectively drain glucose from the microenvironment, resulting in decreased T-effector cell function, which also relies upon glucose oxidation (Chang et al., 2015). Notably, one of the effectors used in this study to promote glycolysis in a tumor line that would otherwise succumb to T-effector surveillance was MYC (Chang et al., 2015). Of course, T-effector cells are just one of a large number of immune cell types present in the tumor microenvironment, and the metabolic reliance of each of them could be effected by either the tumor itself or therapies that specifically target metabolism. The metabolism of other non-tumor cell types beyond the immune compartment are also important to consider with respect to MYC. Indeed, a recent study demonstrated that mice heterozygous for MYC throughout their entire body are smaller, live longer, and are more metabolically active (Hofmann et al., 2015). Thus, targeting MYC-dependent metabolism in cancer could likely impact MYC-dependent metabolism in non-tumor cell types.

## Conclusion

In summary, the role of MYC in the regulation of cancer metabolism is as complex as the diverse functions of MYC itself. What becomes clear after considering the multitude of studies conducted is that the function of MYC, like other oncogenes such as KRAS, is incredibly tissue-specific. However, while the overall metabolic phenotype is usually tissue-specific, dysregulation of individual metabolic pathways are often conserved across tissues, and the combination of these considerations should inform treatment decisions. Cancer research seeks to develop better and potentially curative treatments for MYC driven tumors. Studies of specific oncogene-driven transgenic cancer models allow for discoveries of new metabolic pathways that are deregulated in primary tumors, which could not be otherwise identified in cultured cells. We anticipated that effectively translating findings from studying cancer metabolism and its regulation by oncogenes like MYC or KRAS to the clinic will be accelerated through our understanding of how these oncogenes affect tissue specific metabolism *in vivo*.

## Author contributions

All authors listed, have made substantial, direct and intellectual contribution to the work, and approved it for publication.

### Conflict of interest statement

The authors declare that the research was conducted in the absence of any commercial or financial relationships that could be construed as a potential conflict of interest.
